# Supplementary material for: Post-quantum-inspired scalable blockchain architecture for internet hospital systems with lightweight privacy-preserving access control
Source: PLoS One. 2025 Dec 1;20(12):e0332887. doi: 10.1371/journal.pone.0332887 (PMC12668537; doi:10.1371/journal.pone.0332887)
Supplement: Attachment — Submitted filename: Plos Rebbutal.docx [file pone.0332887.s002.docx]

PONE-D-25-23358

Post-Quantum Scalable Blockchain Architecture for Internet Hospital Systems with Privacy-Preserving Access Control

We extend our sincere gratitude to the Editor of *PLOS ONE* for overseeing the review process of our manuscript titled *“Post-Quantum Scalable Blockchain Architecture for Internet Hospital Systems with Privacy-Preserving Access Control” (PONE-D-25-23358)*. Your time, guidance, and coordination have been instrumental in shaping this work into its present form. For clarity and transparency, **all revisions in the manuscript are marked in red**, while **our detailed responses to reviewer comments in the rebuttal document are provided in** blue. We deeply appreciate your support and the opportunity to revise and improve our submission.

Reviewer #1:

We sincerely thank Reviewer 1 for their thorough and constructive feedback. Your insightful suggestions significantly improved the clarity, technical rigor, and methodological transparency of our manuscript. We have carefully addressed each point and revised the manuscript accordingly, as detailed below.

1. The encryption layer is described as “Kyber-like,” implemented via a mock hybrid scheme. This deviates from an actual Kyber implementation. The authors should clarify which elements of Kyber were preserved (e.g., IND-CPA, key encapsulation) and justify the simulation’s validity. If possible, use standardized libraries (e.g., liboqs) for a more accurate evaluation.

Response: We sincerely thank the reviewer for highlighting the need to clarify the nature of our post-quantum cryptographic implementation. We acknowledge that our original use of the term “Kyber-like” may have inadvertently suggested a complete implementation of the NIST-standard Kyber Key Encapsulation Mechanism (KEM). To address this and ensure full transparency, we have revised both the manuscript content and visual assets accordingly.

Our proposed encryption layer is a Kyber-inspired hybrid simulation, not a full Kyber implementation. Specifically, we preserved the high-level KEM structure and IND-CPA security assumptions, mimicking Kyber’s workflow through randomized symmetric key encapsulation and AES-GCM-based encryption. However, our simulation does not include polynomial ring arithmetic, rejection sampling, or NTT-based polynomial multiplication — elements critical to the standardized Kyber primitive. These exclusions were made to accommodate the constraints of the Google Colab Pro simulation environment while focusing on protocol-level behavior and integration.

To comprehensively address this comment, we made the following key revisions throughout the manuscript:

1. Title: Now reads: “Post-Quantum-Inspired Scalable Blockchain Architecture for Internet Hospital Systems with Lightweight Privacy-Preserving Access Control”, clearly indicating the simulation-based nature.
2. Abstract: We replaced “Kyber lattice scheme” with “Kyber-inspired hybrid simulation model” to remove any implication of direct implementation.
3. Section 1 – Introduction: We now explicitly describe our cryptographic model as a Kyber-inspired hybrid simulation that aims to preserve KEM structure and IND-CPA security guarantees without implementing the full NIST specification.
4. Section 3.1 – System Architecture: In the “Encryption and Access Layer”, we clarified that Kyber is approximated through a hybrid method involving randomized key tokens and symmetric encryption. We emphasize this is not a post-quantum-complete implementation.
5. Section 4.2 – Methodology:
   - We thoroughly revised the subsection to detail the construction of our encryption model.
   - Mathematical formulations for hybrid encryption and decryption are provided (Equations 3 and 4).
   - The rationale behind choosing this approach is explained, particularly in relation to computation resource limits and benchmarking repeatability in the Colab environment.
6. Figures & Visual Assets:
   - All figures referencing “ZkP” or “Kyber” have been updated to reflect “Privacy-Preserving Authorization Proof” and “Kyber-inspired” respectively.
   - Visual representations (e.g., performance graphs, system flowcharts, benchmarking charts) were regenerated in 400–500 DPI with updated legends, axis titles, and figure captions to maintain consistency with the simulation-based terminology.
7. Table 2 – Experimental Configuration:
   - The entry for “Mock Kyber-768” now includes a detailed footnote describing it as a Kyber-inspired hybrid simulation, lacking polynomial ring arithmetic and low-level lattice operations.
   - Authorization proofs are labeled as SHA-256-based non-interactive privacy-preserving commitments, not zero-knowledge constructions.
8. Section 7 – Conclusion: We explicitly mention that our current framework uses a high-level simulation to evaluate cryptographic integration in a scalable IoMT-blockchain system. We have added a forward-looking statement that future iterations will explore full Kyber integration via liboqs-python to enhance cryptographic rigor.

We believe these extensive updates — both in text and supporting materials — fully address the reviewer’s concerns. By clearly distinguishing between simulation and standard implementation, we have improved the transparency, reproducibility, and technical accuracy of our manuscript.

2. The ZkP mechanism is implemented as a simple SHA-256 commitment scheme. This lacks formal zero-knowledge properties and does not meet the standard cryptographic definition of ZkPs. The use of the term “ZkP” should be revised unless replaced with a formal zero-knowledge protocol (e.g., zk-SNARKs or Sigma protocols).

Response: We thank the reviewer for this critical observation regarding our previous use of the term “Zero-Knowledge Proof (ZkP)” in describing our SHA-256-based access control mechanism. We fully acknowledge that our implemented model does not meet the formal cryptographic definition of a ZkP (as in zk-SNARKs, zk-STARKs, or Sigma protocols), and that using the term “ZkP” may have created confusion about the cryptographic rigor and properties of our system.

To address this concern and ensure technical accuracy, we have made extensive revisions across the manuscript, which we summarize as follows:

1. Terminology Corrections Across the Manuscript:
   - All instances of the term “ZkP” have been removed and replaced with accurate alternatives such as:
     - “Privacy-preserving authorization mechanism”
     - “Authorization proof”
     - “Non-interactive access control scheme”
   - These terms better reflect the nature and scope of our implemented SHA-256 commitment-based system and avoid suggesting that it possesses zero-knowledge properties.
2. Section-Specific Revisions:
   - Section 4.3 has been retitled as “Privacy-Preserving Authorization Mechanism” and now includes a clarification that our approach is a SHA-256-based non-interactive commitment scheme, used for lightweight access control without disclosing user secrets. We explicitly state that this is not a formal zero-knowledge proof protocol.
   - Section 6.3’s title has been revised from “Access Control Time (ZkP vs RBAC)” to “Access Control Time (Privacy-Preserving Authorization vs RBAC)”. All descriptions and discussions have also been updated accordingly.
   - Section 6.4, originally “Benchmark: PQC vs. ZkP Operation Times”, has been renamed and rewritten to refer to “Authorization Proof Operations”, clarifying the distinction from formal ZkPs.
   - The Abstract, Introduction, and Conclusion have also been updated to remove ZkP terminology, instead describing the mechanism as a lightweight privacy-preserving access strategy, aligning with its actual properties.
3. Figures and Tables:
   - All figure captions and axis labels (including in performance benchmarks) have been updated to remove “ZkP” and replace it with “Authorization Layer”, “Privacy-Preserving Access Control”, or “Authorization Proof” depending on context.
   - In Table 2 (Experimental Configuration), the relevant section is now labeled “Authorization Layer”, and it includes a footnote clarifying that this is not a cryptographic ZkP, but a commitment-based model for rapid access control simulation.
4. Methodological and Cryptographic Clarification:
   - We now explicitly state in Section 4.3 and Section 7 (Conclusion) that our mechanism lacks zero-knowledge properties, and should not be interpreted as a cryptographically complete ZkP protocol.
   - We further include a commitment that in future work, we intend to integrate formal ZkP schemes, such as zk-SNARKs or Sigma protocols, using standardized libraries (e.g., libsnark, liboqs, or ZoKrates) to enable verifiable, privacy-preserving access control in secure IoMT environments.

We believe these clarifications and corrections resolve the concern thoroughly while still recognizing the practical utility of our current mechanism as a lightweight, scalable alternative for constrained settings such as edge IoT nodes in Internet hospitals. The revised manuscript now accurately reflects both the capabilities and limitations of the scheme and provides a clear roadmap for future cryptographic extensions.

3. Raft is benchmarked against PBFT, but PBFT is a poor baseline at scale. Consider evaluating against HotStuff, Tendermint, or other leader-based consensus mechanisms. Also, clarify the network model—simulating Raft for 1000 nodes under ideal synchronous conditions may not generalize to real-world WAN settings.

Response: We thank the reviewer for this valuable suggestion regarding the benchmarking of Raft against a more scalable and modern set of consensus protocols. We agree that **PBFT alone does not represent a strong comparative baseline**, particularly under large-scale conditions, due to its well-known communication complexity limitations (O(n²)) and lack of scalability in WAN-like networks.

To strengthen the comparative analysis and respond directly to the reviewer’s suggestion, we have made the following substantive updates:

**1. New Consensus Protocol Simulations Added**

We have extended our experimental setup to **benchmark Raft** against two additional leader-based consensus protocols known for their scalability in distributed systems:

- **HotStuff** (D. Y. et al., 2019): A linear communication BFT protocol that is used in systems like Facebook’s Diem blockchain. We implemented a simplified simulation of HotStuff under similar quorum and timeout conditions.
- **Tendermint** (Jae Kwon, 2014): A BFT-based consensus with immediate finality, frequently used in Cosmos-based systems. We simulated its deterministic round-based leader election and gossip-based broadcast for fair comparison.

These additions enable a more balanced and contemporary benchmarking landscape. The **results have been incorporated in Figure 14**, and the **associated discussion is presented in Section 6.6.**

**2. Updated Graphs and Analysis**

We updated our throughput and latency comparison graphs (Figures 14 and 15) to include **Raft, PBFT, HotStuff, and Tendermint** across varying node counts. Key observations include:

- **Raft** remains the most efficient under ideal synchronous conditions, achieving **up to 1.26M TPS** at 1000 nodes.
- **HotStuff** offers strong scalability with lower latency than PBFT, though slightly behind Raft.
- **Tendermint** performs well in latency but is sensitive to node churn due to its deterministic proposer rotation.
- **PBFT**'s performance quickly degrades beyond 100 nodes due to message explosion.

These findings provide a **much clearer picture of protocol trade-offs** under simulated edge-cloud hybrid architectures and back the decision to use Raft in energy- and latency-sensitive IoMT deployments.

**3. Clarified Network Model Assumptions**

In Section 4.4 and again in Section 6.6, we now **explicitly clarify our simulation environment**:

- Our simulations assume a **synchronous network model** with bounded delay and no message loss, consistent with typical assumptions for academic simulation.
- Communication latencies were held constant per protocol instance to isolate consensus efficiency rather than network variability.
- We acknowledge in the manuscript that these assumptions **do not fully represent real-world WAN scenarios**, where asynchrony, churn, and partition tolerance may introduce non-determinism and additional failure modes.

**4. Added Limitations and Future Work**

To ensure transparency, we have updated **Section 7 (Conclusion)** to note the **limitations of synchronous simulations** and explicitly state that real-world WAN performance may vary significantly. We also commit to exploring **asynchronous and adversarial network models** in future studies using event-driven simulation frameworks like NS-3 or PeerSim.

We believe these additions provide a **thorough and technically grounded response** to the reviewer’s concern, expanding the rigor and relevance of our consensus benchmarking while transparently acknowledging simulation constraints.

4. The paper lacks a formal threat model. Clarify what adversarial behaviors are considered (e.g., node compromise, data replay, sensor spoofing), and whether the system ensures confidentiality, integrity, and availability under partial trust assumptions.

Response: We appreciate the reviewer’s observation regarding the absence of a formal threat model. In response, we have now incorporated a dedicated subsection titled **“4.7 Formal Threat Model”** in the revised manuscript. This section rigorously defines adversarial capabilities under a partial trust assumption using formal constructs, including:

- **Definition 1:** Adversarial capabilities such as node compromise, sensor spoofing, and data replay.
- **Definition 2:** System state formulation incorporating sensor inputs, access tokens, keys, hashes, and blockchain state.
- **Theorem 1:** A proof sketch for authorization security based on the one-way of cryptographic hash functions.
- **Lemma 1 & Lemma 2:** These establish guarantees for transaction integrity and availability under majority-honest quorum in the Raft protocol.

5. The energy consumption claims (e.g., 73% reduction) lack methodology. Describe how energy usage was estimated in the Google Colab simulation environment. Include assumptions and instrumentation (e.g., profiling tools, power models).

Response: We thank the reviewer for highlighting this essential concern regarding our energy estimation methodology. In response, we have introduced a dedicated subsection (**Section 4.6: Energy Estimation Methodology**) that explains our simulation-based approach in detail. This includes the operational-level breakdown of energy-consuming components—cryptographic hashing, consensus messaging, and transmission overhead.

We have also included a formal energy estimation model in **Equation (13)** that computes cumulative energy based on the frequency of operations and per-unit energy benchmarks drawn from peer-reviewed sources. All values and assumptions are supported by prior literature, especially the benchmark study by Platt et al. on blockchain consensus energy profiling, now added as **Reference 45**.

These additions clarify how energy was approximated in a simulation environment like Google Colab and ensure that our reported reductions are supported by transparent and reproducible assumptions. All related content has been marked in red for ease of review.

6. The 3-of-5 median filter for sensor correction is a practical design, but needs benchmarking against standard fault-detection methods (e.g., Kalman filters, statistical anomaly detection). Also, clarify how fault injection was simulated and validated.

Response: We thank the reviewer for this insightful comment. We appreciate the recognition of our 3-of-5 median filter for sensor correction as a practical and lightweight approach. However, we agree that benchmarking it against standard fault detection techniques such as Kalman filters and statistical anomaly detection methods is essential for a comprehensive evaluation.

To address this comment, we have made the following additions and revisions to the manuscript:

**1. Benchmarking Against Kalman Filter Added**

In response to this suggestion, we implemented a **standard Kalman filter**-based anomaly detection pipeline for each patient sensor stream, using first-order state estimation for time-series smoothing and outlier removal. We benchmarked the Kalman filter **against the existing 3-of-5 median filter** in terms of:

- **Anomaly Detection Accuracy** (detection rate of injected faults)
- **Sensor Recovery Precision** (corrected value within ±1.0 of ground truth)
- **Computation Time per Reading**

These comparisons are now presented in **Section 6.7 and Figure 16**, with results summarized as follows:

- The **3-of-5 median filter** achieved **92.6% correction accuracy** at 20% fault injection, compared to **89.3% for Kalman filtering**, with much **lower computation time (0.13ms vs. 0.59ms per reading)**.
- Kalman filtering performed better at **smoothing Gaussian noise**, while the **median filter better handled outliers and abrupt spoofing faults**, due to its robustness to non-Gaussian distributions.
- We also note that **Kalman filters require parameter tuning** (e.g., Q and R matrices) for each sensor stream, whereas the median filter is non-parametric and more adaptive under dynamic sensor behavior.

These findings demonstrate that our proposed median filter is not only lightweight but also competitive in detection precision, especially under resource-constrained IoMT settings.

**2. Clarification of Fault Injection and Validation Methodology**

We have **expanded Section 4.5** to provide clear detail on our fault simulation pipeline. Specifically:

- **Sensor readings were generated using a Gaussian profile centered around a patient’s baseline.**
- Faults were randomly injected at pre-defined intervals and percentages (5%, 10%, 20%, 30%) with the following fault types:
  - **Gaussian noise (μ = 0, σ = ±10–15)**
  - **Spoofing values** (additive outliers ±15 to ±25 units)
  - **Missing values** (NaNs injected at random intervals)
- **Ground-truth readings were logged in parallel** to support validation of correction mechanisms.
- The correctness of each filter’s output was computed by comparing the corrected values to the original clean signal with a threshold of ±1.0 margin.

We also added this simulation description to **Table 2 (Experimental Configuration)** to increase reproducibility.

**3. Justification for Using 3-of-5 Median Filter**

We now explicitly state that the 3-of-5 design was selected for:

- **Simplicity and hardware efficiency** on low-power edge devices,
- **Robustness to outliers and spoofed readings** (due to median insensitivity to extremes),
- **No training or tuning required**, unlike probabilistic filters or ML-based anomaly detectors.

These advantages, coupled with its favorable benchmarking results, reinforce the filter’s suitability for real-time medical telemetry scenarios.

**4. Future Work**

As per the reviewer’s suggestion, we added in Section 7 that **future work will explore statistical anomaly detectors** (e.g., Hampel filters, Mahalanobis distance, and autoencoder-based anomaly scoring) for further refinement and hybrid sensor correction pipelines.

We believe these additions significantly enhance the technical depth and clarity of the sensor fault-handling mechanism, while transparently addressing the reviewer’s benchmarking and validation concerns.

7. The ZkP-based access control assumes static authorization tokens generated by patients, but the policy definition and management mechanism are unclear. Detail how permissions are granted, revoked, and audited in practice.

Response: We thank the reviewer for identifying this important gap in our access control discussion. In response, we have made the following substantial additions to clarify the policy lifecycle and operational workflow of our privacy-preserving authorization mechanism:

1. We have introduced two new subsections in the revised manuscript:
   - **Section 4.3.1: Authorization Proof Construction**
   - **Section 4.3.2: Access Control Policy Lifecycle**

These additions clearly explain how patients generate tokens (permissions), how authorization proofs are constructed, and how these policies can be revoked or audited securely.

2. We have also included two new algorithms to enhance reproducibility and operational transparency:
   - **Algorithm 3**: Robust Lightweight Privacy-Preserving Access Verification (already present).
   - **Algorithm 4**: Authorization Proof Construction and Policy Lifecycle Management, which outlines permission grant, revocation, and audit procedures.
3. These sections emphasize:
   - The patient-centric generation of tokens using HMAC constructs.
   - Secure revocation through hash invalidation and token expiry timestamps.
   - Auditable logs of access decisions using blockchain-based immutable entries.

These updates now make the access control framework not only privacy-preserving and lightweight but also verifiable, auditable, and dynamically managed. We hope this comprehensively addresses the reviewer’s concern and improves the clarity and completeness of our contribution.

8. Avoid using terms like “post-quantum,” “ZkP,” and “privacy-preserving” without formal grounding. Either implement recognized cryptographic constructions or qualify that the system uses approximations for simulation purposes.

Response: We appreciate the reviewer’s attention to the rigorous use of cryptographic terminology and agree that precision is critical when referencing well-defined constructs such as post-quantum cryptography (PQC), zero-knowledge proofs (ZkPs), and privacy-preserving access mechanisms. Accordingly, we have revised the manuscript to reflect simulation-oriented and approximation-based terminology more accurately.

Specifically, we have made the following updates:

1. **Terminology Clarifications Throughout the Manuscript**:
   - All instances of **“ZkP”** have been replaced with **“privacy-preserving authorization”** or **“authorization proof mechanism,”** accurately describing our SHA-256-based commitment model as a lightweight simulation rather than a formal ZkP like zk-SNARKs or Sigma protocols.
   - References to **“post-quantum”** have been changed to **“Kyber-inspired”** or **“PQC simulation model”** to acknowledge that the implemented module does not use formal Kyber KEM primitives but simulates its high-level IND-CPA and KEM properties.
   - Where relevant, **“privacy-preserving”** is now clearly tied to context—such as lightweight, non-interactive access checks—without implying full cryptographic anonymity or indistinguishability under chosen access policies.
2. **Section-Level Revisions**:
   - **Section 4.2** explicitly states that the “post-quantum encryption layer” is a simulated hybrid construction based on randomized key tokens and symmetric AES-GCM encryption, not a full Kyber implementation.
   - **Section 4.3** is now titled “Lightweight Privacy-Preserving Access Control,” but it includes a new disclaimer in **4.3.1 and 4.3.2** that no formal ZkP is implemented, and the design is a commitment-based simulation.
3. **Footnotes and Annotations**:
   - A clear footnote has been added to **Table 2 (Experimental Configuration)** clarifying that *“Mock Kyber-768”* refers to a hybrid simulation without polynomial ring arithmetic or NIST-level post-quantum security proofs.
   - Captions and figure labels have been revised to avoid overstated claims and now include qualifiers like *“simulated”*, *“approximated”*, or *“Kyber-inspired”* where applicable.
4. **Future Work Commitments**:
   - In **Section 7 (Conclusion)**, we now explicitly state our intention to adopt formally verified cryptographic libraries (e.g., liboqs, libsnark) in future iterations to evaluate the system under true post-quantum and ZkP conditions.

These changes ensure the manuscript avoids overclaiming and remains grounded in its simulation scope while transparently describing all cryptographic assumptions and limitations.

9. There is limited discussion on how patients define access policies or how policy conflicts are resolved. A more formal policy language or integration with Attribute-Based Encryption (ABE) could enhance flexibility.

Response: We thank the reviewer for highlighting the need for a more formalized discussion around patient access policy specification and conflict resolution. To address this, we have added a new dedicated subsection **4.3.3: Policy Management and Conflict Resolution**, which formally defines how patient-defined access control policies are modeled, verified, and resolved in cases of conflict.

In this subsection, we introduced a mathematical representation of policies as tuples comprising the requester's role, access level, and permitted data scope. We defined a set of policy and provided formal definitions of policy grant conditions using Equations (8), (9), and (10). These equations describe how a data request is matched against patient-defined permissions and how conflict is resolved using predefined precedence rules.

These additions ensure that our authorization model is not only privacy-preserving but also grounded in a verifiable and logically consistent framework. We also clarified that future work may explore more expressive languages (e.g., Attribute-Based Encryption or ABAC models) to enable fine-grained and dynamic policy evaluation.

Reviewer #2:

We sincerely thank Reviewer 2 for the valuable, constructive, and detailed feedback. Your comments have significantly contributed to the clarity, depth, and rigor of our manuscript. In response, we have made substantial revisions—refining the abstract and related work, aligning all claims with the methodology, and ensuring technical precision throughout. We deeply appreciate your insightful suggestions, which helped us improve both the scientific and practical aspects of the proposed framework.

1. Please revise the abstract. Abstract should contain background of the problem, introduction in one and half lines, main contribution, methodology, and impacts of your work.

Response: We thank the reviewer for pointing out the need to revise the abstract for clarity and completeness. In response, we have comprehensively rewritten the abstract to ensure it follows the recommended structure. The new version includes:

- A brief background introducing the emergence of Internet hospital systems and the associated challenges;
- A clear description of the four primary contributions of our work, including cryptographic innovation, access control, consensus scalability, and sensor correction;
- A summary of the implementation environment and evaluation methodology;
- A detailed impact statement reflecting real-world applicability in terms of performance gains, energy efficiency, and scalability.

These changes have been incorporated into the revised abstract and can be found at the beginning of the manuscript.

2. Literature review is very weak, please revise it accordingly.

Response: We sincerely thank the reviewer for their valuable feedback regarding the depth of our literature review. In response, we have carefully revised the *Related Work* section to present a more structured and comprehensive analysis of relevant prior studies. We now emphasize the methodologies, strengths, and limitations of each work in greater detail, with a clearer connection to the gaps our system addresses. Additionally, we have included a dedicated comparative table (Table 3) to summarize and contrast the most relevant blockchain-based healthcare frameworks. Finally, we have added a new concluding paragraph (highlighted in red) that contextualizes the unique contribution of our proposed framework relative to existing approaches. These updates improve the scholarly rigor and relevance of our literature review section.

3. Research Methodology is still imbigues, clearly re write and also present it visually.

Response: We sincerely thank the reviewer for their constructive feedback, which has helped us substantially improve the quality and clarity of our manuscript. In response to your suggestions, we have made several significant enhancements throughout the paper:

1. **Revised Abstract:** We have restructured the abstract to better reflect the problem background, concise introduction, methodological overview, key contributions, and real-world impact of our work (see updated Abstract section).
2. **Expanded Literature Review:** The *Related Work* section has been substantially strengthened with additional studies, clearer methodological contrasts, and a new comparative summary table (Table~\ref{tab:related_work_comparison}) that outlines the strengths and limitations of relevant frameworks. A red-highlighted concluding paragraph now better positions our contribution relative to prior art.
3. **New Sections and Methodological Details:**
   - We have added several new technical subsections:
     - Section 4.3.1: **Authorization Proof Construction**
     - Section 4.3.2: **Access Policy Assignment and Revocation**
     - Section 4.3.3: **Attribute-based Policy Derivation**
     - Section 4.6: **Energy Consumption Estimation Methodology**
     - Section 4.7: **Formal Threat Model**
   - Each subsection includes algorithmic or mathematical formulations where appropriate to clarify and rigorously define system behavior.
4. **Updated Figures and Architecture Diagram:** Figures 1 and 2 have been revised to reflect architectural updates, including more accurate representations of the Kyber-inspired encryption module and the lightweight authorization layer.
5. **Terminology Clarifications:**
   - Following your suggestion and Reviewer 1’s comments, we have updated all occurrences of ambiguous terms like “ZkP” and “post-quantum” to accurately describe our implemented simulations, rather than implying full cryptographic formality.
   - Appropriate clarifying notes are now included in Section 1 (Introduction), Section 4 (Methodology), and the Experimental Configuration table caption.

These changes collectively improve the scientific validity, transparency, and overall readability of our manuscript. All major additions are marked in **red** for your convenience. We are grateful for your insightful recommendations, which have significantly enriched the final version.

4. Align your claims with methodology and abstract.

Response: We thank the reviewer for this important observation. In response, we have thoroughly reviewed the manuscript to ensure that all claims made in the abstract, introduction, and conclusion are fully supported by the detailed methodology, algorithms, equations, and experimental results presented in the main text. Specifically:

- The abstract now clearly reflects the use of **Kyber-inspired hybrid encryption**, **lightweight privacy-preserving authorization**, **Raft consensus**, and **median-based sensor correction**, all of which are elaborated upon in Sections 4.2–4.4.
- Performance metrics reported in the abstract (e.g., latency reductions, energy savings, accuracy) are now directly traceable to their respective experimental discussions in Section 6, with assumptions and simulation tools detailed in Section 4.6.
- Claims regarding security guarantees are now grounded in our newly added **Formal Threat Model** (Section 4.7), which defines adversarial behaviors and system guarantees using formal theorems and lemmas.

All corresponding revisions are marked in **red** for transparency. These changes ensure consistency between the narrative and technical content and improve the manuscript’s integrity.
