# Supplementary material for: Post-quantum-inspired scalable blockchain architecture for internet hospital systems with lightweight privacy-preserving access control
Source: PLoS One. 2025 Dec 1;20(12):e0332887. doi: 10.1371/journal.pone.0332887 (PMC12668537; doi:10.1371/journal.pone.0332887)
Supplement: Attachment — Submitted filename: PONE-D-25-23358R1-Rebuttal.docx [file pone.0332887.s003.docx]

**Manuscript Title:**
*Post-Quantum-Inspired Scalable Blockchain Architecture for Internet Hospital Systems with Lightweight Privacy-Preserving Access Control*

**Manuscript ID:**
PONE-D-25-23358R1

Dear Dr. Gauhar Ali, Academic Editor,

We sincerely thank you and the reviewers for your careful consideration of our manuscript and for providing constructive feedback that has significantly improved the quality and clarity of our work. In the revised submission, all changes to the manuscript text are highlighted in **red** for easy identification. In this rebuttal letter, our detailed point-by-point responses to the reviewer comments are presented in **blue** for clarity.

We are grateful to the reviewers for their thoughtful and constructive comments, which helped us refine the framing of our methodology, ensure consistency of reported results, clarify authorization and key management details, and add a dedicated limitations section to contextualize our contributions. We believe these revisions have strengthened the manuscript considerably.

We once again thank the Academic Editor and reviewers for their valuable time and efforts in improving our paper.

**Reviewer’s Comments and responses:**

1. Methodology framing. The work relies on a simulated “Kyber-inspired” scheme and assumes a synchronous, constant-delay network. It should be clearly emphasized that the findings are derived from simulation rather than real-world post-quantum cryptographic deployments or production consensus systems.

**Response:** We thank the reviewer for highlighting this important point. In the revised manuscript, we have explicitly emphasized that the work is simulation-based and does not rely on real-world PQC deployments or asynchronous consensus networks. The following changes were made:

- **Abstract:** We now state that the framework is “simulation-based,” and that the cryptographic layer uses a Kyber-inspired hybrid encryption simulation under a synchronous constant-delay network assumption.
- **Section 4 (Methodology introduction):** We added a clarification that all results are derived from controlled simulations, with Kyber modeled as a simulated hybrid encryption and consensus tested under a synchronous constant-delay network.
- **Section 4.2 (Encryption Layer):** We clarified that the model does not implement full polynomial ring arithmetic or official Kyber primitives but instead approximates performance characteristics.
- **Section 4.4 (Scalable Raft-Based Consensus):** We added a note that all consensus benchmarks were performed under synchronous, constant-delay network assumptions and do not capture WAN packet loss or asynchrony.

These changes ensure that the simulation context and modeling assumptions are clear to readers.

2. Numerical consistency. Several headline claims conflict with reported results (e.g., “73% energy savings” vs ~9–12%, “4× memory reduction” vs ~14%, “0.4 ms verification” vs ~0.002 ms). These discrepancies should be resolved so that the abstract and body consistently reflect the actual measurements.

**Response:** We thank the reviewer for identifying these inconsistencies. We carefully revised the manuscript to ensure that all headline performance numbers are consistent with the detailed results presented in the Results section. The following corrections were made:

- **Abstract:** Verification latency corrected to ~0.002 ms, energy savings revised to ~9–12%, and memory reduction corrected to ~14%.
- **Section 6.1 (Access Control Time):** Verification latency consistently reported as ~0.002 ms.
- **Section 6.7 (Memory Usage for Encrypted Records):** Memory usage reduction consistently reported as 14.3%.
- **Section 6.12 (Energy Efficiency Observation):** Energy savings consistently reported as 9.4–12.3%.
- **Section 7 (Conclusion and Future Work):** All performance summaries updated to match the corrected results.

These revisions eliminate all discrepancies between the abstract, body, and conclusion, ensuring that the manuscript now presents a consistent and accurate representation of the experimental findings.

3. Consensus configuration. The experimental setup for PBFT and other protocols is inconsistent across sections (e.g., 20 vs 1000 nodes). Baselines should be aligned under uniform conditions such as node count, quorum thresholds, and block sizes to ensure fair comparison.

**Response:** We thank the reviewer for this valuable observation. In the revised manuscript, we have clarified why PBFT was only simulated with 20 nodes, while Raft, HotStuff, and Tendermint were evaluated with up to 1000 nodes.

- **Section 5.5 (Consensus Protocols):** We now explicitly state that PBFT was restricted to 20 nodes due to its $O(N^2)$ communication complexity, which made large-scale simulations computationally infeasible in our environment.
- **Section 6.2 (Consensus Benchmarking):** We added a clarification that PBFT latency trends at larger scales are extrapolated from the literature and from its theoretical complexity, rather than from direct 1000-node simulations.
- **Section 6.3 (Consensus Latency Benchmark):** We further emphasized that PBFT results represent small-scale baselines only, whereas Raft, HotStuff, and Tendermint serve as the true scalability benchmarks.

These clarifications ensure transparency regarding experimental design and help readers interpret PBFT’s role as a comparative baseline, while making clear that Raft, HotStuff, and Tendermint are more representative for large-scale deployments.

4. Authorization and security details. The description of the authorization model leaves ambiguity around replay protection and key management. Explicitly specify how freshness (e.g., nonces or timestamps) is enforced and clarify how HMAC-based policies are verified without insecure key sharing.

**Response:** We thank the reviewer for raising this important point. In the revised manuscript, we have expanded the Access Control section to explicitly describe both replay protection and secure key management:

- **Section 4.3.2 (Authorization Proof Construction):** We added that each authorization proof now incorporates a nonce and a timestamp. The verifier checks that the timestamp is within an acceptable validity window and that the nonce has not been previously used. This mechanism ensures freshness and prevents replay attacks.
- **Section 4.3.3 (Policy Management and Conflict Resolution):** We clarified that HMAC keys are generated locally on the patient’s device and never shared directly with other nodes. Only their commitments are recorded on-chain. Keys can be rotated periodically, or when a policy is updated, to provide forward secrecy and prevent long-term exposure of any single key.

These additions remove ambiguity, ensure resistance to replay attacks, and demonstrate that HMAC-based verification does not rely on insecure key sharing.

5. Limitations statement. The paper should include a dedicated limitations section highlighting the reliance on synthetic IoMT data, mock cryptographic implementations, synchronous network assumptions, optimistic TPS estimates, and varying fault-rate assumptions. Acknowledging these constraints will properly contextualize the contributions.

**Response:** We sincerely thank the reviewer for this constructive suggestion. In the revised manuscript, we have added a dedicated subsection **“Limitations”** before the Conclusion. This section acknowledges the key assumptions of our study, including the use of synthetic IoMT data, the reliance on a Kyber-inspired simulation rather than a certified PQC implementation, the adoption of a synchronous constant-delay network model, optimistic TPS estimates under controlled settings, the restriction of PBFT simulations to 20 nodes due to quadratic complexity, and the simplified fault injection assumptions for IoMT devices. We framed these points not as weaknesses but as necessary design choices for controlled experimentation, and we emphasized that they open up avenues for future work in real-world deployments. By clearly stating these limitations, we aim to provide transparency and proper context for our contributions.
